# Effects of carcinogen and cortisone on mast cells in the hamster cheek pouch.

**DOI:** 10.1038/bjc.1967.20

**Published:** 1967-03

**Authors:** C. J. Smith

## Abstract

**Images:**


### EFFECTS OF CARCINOGEN AND CORTISONE ON MAST CELLS IN THE HAMSTER CHEEK POUCH

C. J. SMITH

*From the Department of Dental Science, Royal College of Surgeons of England, Lincoln's Inn Fields, London, W.C.2*

Received for publication October 28, 1966

MAST CELLS have been reported to be prominent in the stroma of premalignant epithelial lesions of human skin (Cawley and Hoch-Ligeti, 1961), and of cervix (Dunn and Montgomery, 1957 ; Graham and Graham, 1966) but to be reduced in the vicinity of malignant tumours (Cawley and Hoch-Ligeti, 1961 ; Dunn and Montgomery, 1957 ; Graham and Graham, 1966 ; and Lascano, 1958). Variations in mast cell population have also been produced in tissues exposed to chemical carcinogens ; Cramer and Simpson (1944) described an increase in mast cells related to the development of epithelial hyperplasia in response to painting mouse skin with methylcholanthrene. This increase occurred long before the development of carcinoma, from which the mast cells were almost completely absent. Similar results have been described by Morris (1957), Fiore-Donati *et al.* (1962), and Chieco-Bianchi *et al.* (1963).

Asboe-Hansen and Zachariae (1955) found that regression of carcinogen-induced papillomas could be brought about by treatment with cortisone, various changes in the connective tissue mast cells being associated with regression. There is other evidence that cortisone has an effect upon mast cells, with regard to both distribution and individual morphological characteristics (Wegelius and Asboe-Hansen, 1956 ; Cavallero and Braccini, 1951 ; Asboe-Hansen, 1952 ; Fulton and Maynard, 1953). These features suggest that mast cells may be intimately associated with the neoplastic process, or resistance to it, and that neoplasia may be influenced by cortisone acting through the medium of mast cells.

This communication describes a study of the mast cell population in the tissues of the hamster cheek pouch during chemical carcinogenesis, together with an account of the modification of this reaction during simultaneous treatment with cortisone acetate.

### MATERIALS AND METHODS

*Experimental carcinogenesis.*—Fifty golden Syrian hamsters (*Mesocricetus auratus*), between 4 and 6 weeks of age, were divided into two equal groups (A and B) each containing almost equal numbers of either sex. All the animals received regular applications of the same carcinogen to one of their cheek pouches while the contralateral pouch remained unpainted. One of the groups of twenty-five animals (Group A) received subcutaneous injections of an aqueous solution of cortisone acetate (" Cortisyl ", Roussel Laboratories Ltd.) into the skin of the back. Each injection contained 2·0 mg. cortisone acetate and was given immediately before each treatment of the cheek pouch. The other group (Group B) received no treatment other than carcinogen applications to one pouch. The carcinogen employed was a 0·5% solution of 7,12-dimethyl(α)benzanthracene in liquid paraffin, applied three times each week with a sable-hair paint brush.

A further group of sixteen hamsters (Group C), of the same age and strain as the other two groups, received thrice weekly applications of the same carcinogen to one pouch, while the contralateral pouch was painted with the pure liquid paraffin vehicle at the same frequency. Thus mast cells could be studied in : normal pouches (Group B) ; paraffin painted pouches (Group C) ; carcinogen painted pouches (Groups B and C) ; normal pouches from cortisone treated animals (Group A) ; and carcinogen painted pouches from cortisone treated animals (Group A).

*Examination of mast cells.*—Samples of pouches receiving each type of treatment were obtained at regular weekly intervals during the experimental period. Pouches were dissected out immediately after death by stunning, and divided so that half could be fixed in 10% formal saline for routine histological procedures while the remainder was frozen with solid carbon dioxide and sectioned on a cryostat for histochemical investigation. Mast cells were displayed in cryostat sections by the methyl green-pyronin Y method (Kurnick, 1955) and toluidine blue method (Pearse, 1960), and in paraffin embedded sections by the Luna (Ambrogi, 1960), Dominici (Ambrogi, 1960) and standard toluidine blue (Pearse, 1960) methods. All of these techniques allowed easy identification of individual mast cells and gave a good indication of the state and appearance of the metachromatic granules.

### RESULTS

Few mast cells are present in the normal hamster cheek pouch, and these are confined almost entirely to loose perivascular connective tissue deep to the muscle layer (Fig. 1). Individual cells are characteristically packed with metachromatic granules of regular shape and size (Fig. 2). Occasionally, similar granules are found in the tissue immediately surrounding a mast cell, but these are few in number.

Painting the cheek pouches with liquid paraffin produces no gross or microscopic changes in the epithelial surface. There are, however, a few minor alterations in the mast cells, principally a slight initial increase in numbers which subsides to normal within three or four weeks, and also a slight increase in extracellular granulation with a correspondingly slight decrease in intracellular granules.

Animals receiving regular subcutaneous doses of cortisone acetate also have a mast cell pattern at variance with the normal, but this difference does not become evident until eight weeks, or more, have elapsed. Just preceding this period there is a short episode of excessive extracellular granulation which quickly subsides and is followed by a decrease in the expected number of mast cells in the tissues. Most of the mast cells retain a normal intracellular complement of granules, but some show a reduction.

The progress of carcinogenesis has been reported previously (Camilleri and Smith, 1964) and closely follows that originally described by Salley (1954) and Morris (1957). An inflammatory reaction occupies the first two or three weeks and then subsides into a latent period. Papillomas appear after the eighth week, and later develop into malignant tumours, from the twelfth week. During the initial inflammatory reaction, the mast cells increase in number and become more widely distributed, some being found in the muscle layer while others have penetrated still further and are seen in the subepithelial connective tissue (Fig. 3). They are no longer strictly localised to the environs of blood vessels, although there is an accompanying increase in vascularity. A few mast cells are smaller than normal and this may partly explain the observation that their cytoplasm is even more densely packed with metachromatic granules than usual. Extracellular granules can be found but with no greater frequency than around normal mast cells. The occasional cell which exhibits only a few intracellular granules is probably a macrophage eliminating some of the extracellular granules (Fig. 4). These cells are more evident in the deep connective tissue at the height of the inflammatory response. Resolution of the inflammatory response leaves a pouch of normal appearance even though painting with carcinogen continues. From the eighth week of painting, epithelial hyperplasia is evident however, and is accompanied by a small increase in mast cell numbers with a more widespread distribution than normal (Fig. 5) and a slight increase in extracellular granulation. Papillomas that develop from regions of epithelial hyperplasia contain few mast cells (Fig. 6), though the deep connective tissue below the pedicle usually contains a large number of these cells (Fig. 7). Precancerous epithelium is not associated with a conspicuous increase in mast cells in the underlying connective tissue, and areas where malignant invasion has occurred remain entirely free from mast cells (Fig. 8). It was noteworthy that, as carcinogenesis progressed, sections stained with toluidine blue, which is also present in Dominici's stain, demonstrated mast cells with increasing intensity.

When carcinogen is applied to the pouches of animals receiving simultaneous injections of cortisone acetate most of the changes occurring among mast cells are similar to those in pouches receiving only carcinogen treatment, except for more marked extracellular granulation. Later however, the appearance more closely resembles that in animals receiving injections of cortisone acetate only, there being a reduction in number of mast cells from about the eighth week of

---

EXPLANATION OF PLATES

FIG. 1.—Normal hamster cheek pouch showing normal distribution of mast cells (MC) in deep connective tissue (dct). Epithelium (e), subepithelial connective tissue (sct) and muscle (m) are free from mast cell infiltration. Toluidine blue. × 70.

FIG. 2.—Mast cells in normal hamster cheek pouch, packed with metachromatic granules and also showing occasional extracellular granules. Toluidine blue. × 275.

FIG. 3.—Carcinogen-treated hamster cheek pouch during initial inflammatory reaction. Compare with Fig. 1. Mast cells now infiltrating muscle layer and entering subepithelial connective tissue which contains many inflammatory cells. Toluidine blue. × 70.

FIG. 4.—Mast cells during initial inflammatory reaction, same section as Fig. 3. Compare with Fig. 2. Metachromatic granules depleted in some cells which may be macrophages. Toluidine blue. × 275.

FIG. 5.—Carcinogen-treated hamster cheek pouch showing mild epithelial hyperplasia (e). Mast cells (MC) widely dispersed throughout subepithelial connective tissue and muscle layer but with no sites of aggregation. Luna's stain. × 65.

FIG. 6.—Papilloma from carcinogen-treated hamster cheek pouch. Connective tissue core (ct) free from mast cell infiltration but pedicle (p) contains a dense collection of mast cells shown at high magnification in Fig. 7. Luna's stain. × 22.

FIG. 7.—Higher magnification of pedicle region from papilloma shown in Fig. 6 to demonstrate dense aggregation of mast cells. Luna's stain. × 125.

FIG. 8.—Area of squamous cell carcinoma from carcinogen-treated hamster cheek pouch. The connective tissue stroma is completely free from mast cell infiltration. Luna's stain. × 65.

Smith.

Smith.

the experiment. Extracellular granulation is most evident in the group receiving both cortisone and carcinogen, but no compensatory reduction of intracellular granules is apparent. No evidence has been obtained to support opinions that cortisone affects carcinogenesis in such a way as to produce earlier invasion, or more aggressive tumours, or more widespread metastases.

### DISCUSSION

One of the earliest reports of the behaviour of mast cells under the influence of carcinogens was given by Orr (1938). He showed that the mast cell population of the skin in mice increased in response to painting with a carcinogenic substance for three weeks or more. In a similar experiment Fiore-Donati *et al.* (1962) described the mast cell reaction as becoming evident 15–20 days after painting with DMBA and increasing progressively until papillomas appeared after 39 days ; the mast cells were small, sparsely granulated and poorly metachromatic. Hamsters were found to respond in a similar way to skin painting (Chieco-Bianchi *et al.*, 1963), their accumulating mast cells being smaller and less granulated than normal hamster mast cells and located mostly beneath the hyperplastic epithelium. Morris (1957) studied the distribution of mast cells during hamster cheek pouch carcinogenesis but did not describe an increase in mast cell population during the initial inflammatory reaction. An increase was found, however, in the early stages of the present experiment and this was apparently similar to the early increases found by Orr, by Fiore-Donati *et al.*, and by Chieco-Bianchi *et al.* But this increase was not gradually progressive, like that described by Fiore-Donati *et al.*, for the response evoked during the initial inflammatory reaction subsided rapidly and a normal appearance was maintained until the onset of epithelial hyperplasia.

Variations in individual mast cells in the present study were found to be more extensive than the smaller and less granular cells described by Fiore-Donati *et al.* and Chieco-Bianchi *et al.* Although this type of mast cell was occasionally seen, the most frequent departure from the normal was a small cell with dense cytoplasmic granulation. These morphological changes in the initial inflammatory stage were accompanied by a dispersion of mast cells throughout the tissues more widespread than that seen under normal conditions.

The initial mast cell response to a carcinogenic hydrocarbon was shown by Orr to be nearly equalled by the response to a non-carcinogenic hydrocarbon. However, in the present experiment painting cheek pouches with the saturated non-carcinogenic hydrocarbon liquid paraffin caused only a slight increase in number of tissue mast cells.

An association between accumulation of mast cells and epithelial hyperplasia induced by a carcinogen had first been described by Cramer and Simpson (1944) in their experiments with methylcholanthrene on mouse skin. This increase occurred long before the development of carcinoma, from which the mast cells were completely absent. They also suggested a correlation between resistance to the development of tumours and intensity of reaction, the mast cells being more numerous in the animals with greatest resistance, and less numerous in animals with weakest resistance. Morris (1957) found a marked increase in mast cells during hamster cheek pouch carcinogenesis at the stage of epithelial hyperplasia, with a more diffuse distribution than that found in normal cheek pouch.

There were practically no mast cells around areas of malignant invasion. A similar result was obtained in the present experiment. Also, the increase in mast cell population was not as pronounced during epithelial hyperplasia as that described in skin carcinogenesis, nor was it as heavy as the similar reaction in the initial inflammatory stage.

The observations on experimental material compare with an increased number of mast cells found in the contiguous tissues of human skin cancer (Cawley and Hoch-Ligeti, 1961), and a deficiency of mast cells in the stroma of malignant tumours (Lascano, 1958). In a study of the premalignant stages leading to cervical cancer in the human, Dunn and Montgomery (1957) found the greatest increase in mast cells to occur in carcinoma-*in-situ*, there being a marked reduction at the onset of invasion. More recently, Graham and Graham (1966) have confirmed these observations and, in addition, have found that response to treatment is better in those patients with an initial high mast cell count in the stroma. It remains obscure, however, whether the mast cells accumulate in order to slow the rate of growth of the tumour, or because the rate of tumour growth is slow.

Although the work of Graham and Graham indicates that the extent of mast cell infiltration in the stroma may have prognostic significance, from the experiments performed in this study it is clear that transient variations occurring during carcinogenesis in the hamster cheek pouch form no basis for the early recognition of impending malignancy.

Corticotrophin and cortisone acetate both caused hamster cheek pouch mast cells to become degranulated (Wegelius and Asboe-Hansen, 1956), with clumping of the remaining granules and vacuolation of cytoplasm ; such changes were less marked away from the site of injection. Similar effects had been previously described for rats (Cavallero and Braccini, 1951) and for humans (Asboe-Hansen, 1952) in both of which an irregularity in the size of mast cells was observed. More closely related to the method used in the present investigation, Fulton and Maynard (1953) injected hamsters subcutaneously with cortisone acetate (5 mg. per day for 12–23 days) and observed mast cell counts in the cheek pouch. They reported a definite decrease in number, the remaining mast cells often being spindle shaped, and frequently adjacent to extracellular granules and amorphous stainable material. The reduction in number was noticeable especially in the tissues remote from blood vessels. In the present experiment the main difference between hamsters receiving cortisone and normal controls, and this only after eight weeks of treatment, was a short period of excessive extracellular granulation followed by a decrease in the number of mast cells. Some of these also showed a decrease in granular contents. These changes are similar to those previously reported, though the degree of alteration has probably been reduced by the smaller dosage and remote site of injection.

The only extra effect observed in the carcinogen-treated animals given cortisone was a slight enhancement of the extracellular granulation without, however, a corresponding loss of intracellular granular substance. The increase in mast cells during the initial inflammatory reaction still occurred but there was a decrease, instead of an increase, at the period of epithelial hyperplasia. This did not seem to alter the malignant potential of the tissue, for malignant tumours developed in the same time and in the same manner as in animals not receiving cortisone. No regression of hamster cheek pouch tumours was observed, unlike the results of Asboe-Hansen and Zachariae (1955) that injections of hydrocortisone beneath carcinogen-induced papillomas on mouse skin caused regression. However, in the present experiment the cortisone acetate was injected subcutaneously at a site remote from the tumours.

The changes that have been observed in mast cells are related to four main properties, number, site, morphology and extracellular granulation, which will now be further discussed. In normal tissues occasional extracellular granules are observed in the vicinity of mast cells ; these may be ascribed to the effects of normal turnover of cells and to manipulation of the tissues. The slight increase observed in pouches painted with liquid paraffin is probably attributable to the greater degree of minor trauma, compared to unpainted pouches, to which they had been subjected. It would seem, however, that the effect is only transitory because the appearance of the mast cells soon returns to normal. During carcinogenesis the degree of extracellular granulation is hardly altered; seemingly neither the carcinogen nor liquid paraffin affects the cytoplasmic granules. It appears to be probable that the effect of cortisone in producing more extracellular granules is enhanced by the simultaneous application of carcinogen to the pouch ; however, this has no significance with regard to the ensuing development of malignant tumours.

Fawcett (1955) warned against ascribing specific effects upon mast cells to various agents, particularly if they are administered in a hypotonic solution, if they are damaging to cell membranes, or if they are surface active agents. These will all cause extracellular granules to appear in the vicinity of mast cells ; where an increase has been seen in the present study it is possible to see one or more of these factors at work. Smith and Lewis (1958), Higginbotham, Dougherty and Jee (1956) and Fawcett (1955) have shown that such extruded granules are taken up by macrophages and fibroblasts in the vicinity so that, if a single stimulus has caused extrusion of granules, none is found extracellularly after 24 hours. Some of the phagocytes, however, contain so many granules that they resemble normal mast cells ; such granules are soon destroyed. Failure to account for phagocytes containing metachromatic material, and including them as mast cells, obviously leads to errors in interpreting data involving changes in mast cell numbers. Smith and Lewis described a large increase in numbers of mast-like cells, which in fact were granule-containing macrophages and fibroblasts, in the cheek pouches of hamsters after treatment with cortisone, ACTH or X-irradiation. It is therefore extremely difficult to decide whether the increase in mast cells in any given tissue that also shows fluctuation in extracellular granulation is real or only apparent. Any morphologically atypical mast cell may in fact be a macrophage or fibroblast that has taken up metachromatic granules. Apart from this giving rise to an apparent increase in numbers of tissue mast cells, there may also be a real increase in numbers by recruitment from other sites or by increased differentiation. A real increase may, of course, occur in isolation. Differences in appearance of individual mast cells may also be explained on the basis of age. Fawcett (1955) described how regeneration of cells appeared to take place from spindle shaped cells in the adventitia of small blood vessels that develop granules with typical staining properties ; these cells became more numerous and gradually spread out into the tissues. Riley (1953) found mast cells with two distinct appearances ; one of these stained densely orthochromatically and was found chiefly in the adventitia of vessels with muscle coats, the other type were larger, filled with metachromatic material and found mainly around capillaries and free in tissue spaces. The first type described was considered to be an early form, the latter type developing from them.

A decrease in individual mast cell granulation, concomitant with an increase in mast cell population, was described by Sylvén and Larsson (1948) as occurring upon painting mouse skin with methylcholanthrene dissolved in benzene. Maximum depletion of granular substance was reached in 3–5 days and a normal level was restored after 6 days.

Experiments in organ culture led Chayen, Darracott and Kirby (1966) to suggest that increases in population of mast cells may be apparent rather than real. They suggest that histamine released from damaged tissue serves to disclose pre-existing mast cells, and they re-interpret the role of the mast cell as responding to elevated levels of tissue histamine and possibly " detoxicating " histamine rather than ejecting it. If this theory were correct, the presence of histamine—at least during the inflammatory stage of carcinogenesis—could account for the observed increase in numbers of mast cells, and this would be attributable to improved disclosure rather than a real increase. It is, however, not clear whether the authors of this theory took into account the possibility that an increase in mast cell numbers may be simulated by phagocytosis of extruded granules by macrophages and fibroblasts.

With regard to altered distribution of mast cells, it is apparent that this may not, in fact, involve mast cells at all. Cells found remote from the conventional mast cell sites may be wandering phagocytes containing metachromatic granules ; or they may be mast cells newly disclosed by the presence of histamine ; or they may be mast cells truly dispersed from their usual sites.

The increasing intensity of staining of mast cells by toluidine blue during carcinogenesis cannot be explained in the light of present knowledge of the mechanism of action of this stain.

### SUMMARY

Alterations in morphology and distribution of mast cells have been found to occur during hamster cheek pouch carcinogenesis with 7,12-dimethyl($\alpha$)benzanthracene. In the initial inflammatory reaction there is an increase in mast cell numbers, an extension of their normal distribution, and variation in the granular contents of the cells. Though there is a return to normal when the inflammatory reaction resolves, further changes are seen to accompany epithelial hyperplasia. Again the mast cells are more widely distributed than normally but no particularly heavy aggregations have been found beneath premalignant epithelium. The pedicle region of papillomas, however, does seem to contain a large number of mast cells of normal appearance, but the stroma of squamous cell tumours, which are the end result of carcinogen treatment, remains completely free from mast cell infiltration.

Cortisone acetate given subcutaneously to the hamsters produces slight alterations in the mast cell population of the cheek pouches, whether these are being treated with carcinogen or not.

I would like to express my thanks to Professor B. Cohen for his advice and encouragement during this investigation which was carried out during the tenure of a Medical Research Council Scientific Assistantship and a Prophit Studentship in Cancer Research.

### REFERENCES

AMBROGI, L. P.—(1960) 'Manual of Histologic and Special Staining Technics'. 2nd edition. London (The Blakiston Division, McGraw-Hill Book Co. Inc.).

ASBOE-HANSEN, G.—(1952) *Proc. Soc. exp. Biol. Med.*, **80**, 677.

ASBOE-HANSEN, G. AND ZACHARIAE, L.—(1955) *Acta path. microbiol. scand.*, **37**, 145.

CAMILLERI, G. E. AND SMITH, C. J.—(1964) *Acta cytol.*, **8**, 85.

CAVALLERO, C. AND BRACCINI, C.—(1951) *Proc. Soc. exp. Biol. Med.*, **78**, 141.

CAWLEY, E. P. AND HOCH-LIGETI, C.—(1961) *A.M.A. Archs Derm.*, **83**, 92.

CHAYEN, J., DARRACOTT, S. AND KIRBY, W. W.—(1966) *Nature, Lond.*, **209**, 887.

CHIECO-BIANCHI, L., FIORE-DONATI, L., PENNELLI, N. AND BERTACCINI, G.—(1963) *Nature, Lond.*, **199**, 293.

CRAMER, W. AND SIMPSON, W. L.—(1944) *Cancer Res.*, **4**, 601.

DUNN, M. R. AND MONTGOMERY, P. O'B.—(1957) *Lab. Invest.*, **6**, 542.

FAWCETT, D. W.—(1955) *Anat. Rec.*, **121**, 29.

FIORE-DONATI, L., DE BENEDICTIS, G., CHIECO-BIANCHI, L. AND BERTACCINI, G.—(1962) *Nature, Lond.*, **193**, 287.

FULTON, G. P. AND MAYNARD, F. L.—(1953) *Proc. Soc. exp. Biol. Med.*, **84**, 259.

GRAHAM, R. M. AND GRAHAM, J. B.—(1966) *Surgery Gynec. Obstet.*, **123**, 3.

HIGGINBOTHAM, R. D., DOUGHERTY, T. F. AND JEE, W. S. S.—(1956) *Proc. Soc. exp. Biol. Med.*, **92**, 256.

KURNICK, N. B.—(1955) *Stain Technol.*, **30**, 213.

LASCANO, E. F.—(1958) *Cancer, N.Y.*, **11**, 1110.

MORRIS, A. L.—(1957) 'Studies on the production and histochemistry of experimental oral cancer in the hamster'. Ph.D. thesis, University of Rochester.

ORR, J. W.—(1938) *J. Path. Bact.*, **46**, 495.

PEARSE, A. G. E.—(1960) 'Histochemistry: Theoretical and Applied'. 2nd edition. London (J. & A. Churchill Ltd.).

RILEY, J. F.—(1953) *J. Path. Bact.*, **65**, 461.

SALLEY, J. J.—(1954) *J. dent. Res.*, **33**, 253.

SMITH, D. E. AND LEWIS, Y. S.—(1958) *Anat. Rec.*, **132**, 93.

SYLVÉN, B. AND LARSSON, L. G.—(1948) *Cancer Res.*, **8**, 449.

WEGELIUS, O. AND ASBOE-HANSEN, G.—(1956) *Acta endocr., Copenh.*, **22**, 157.


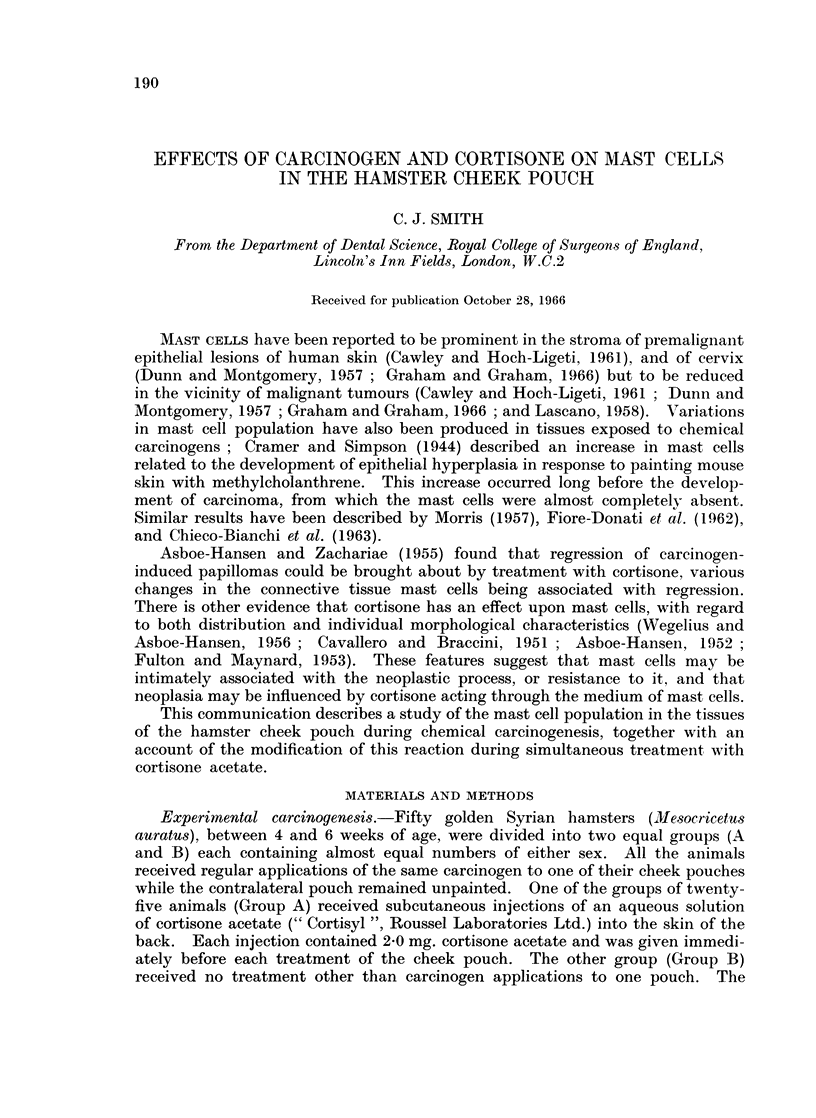

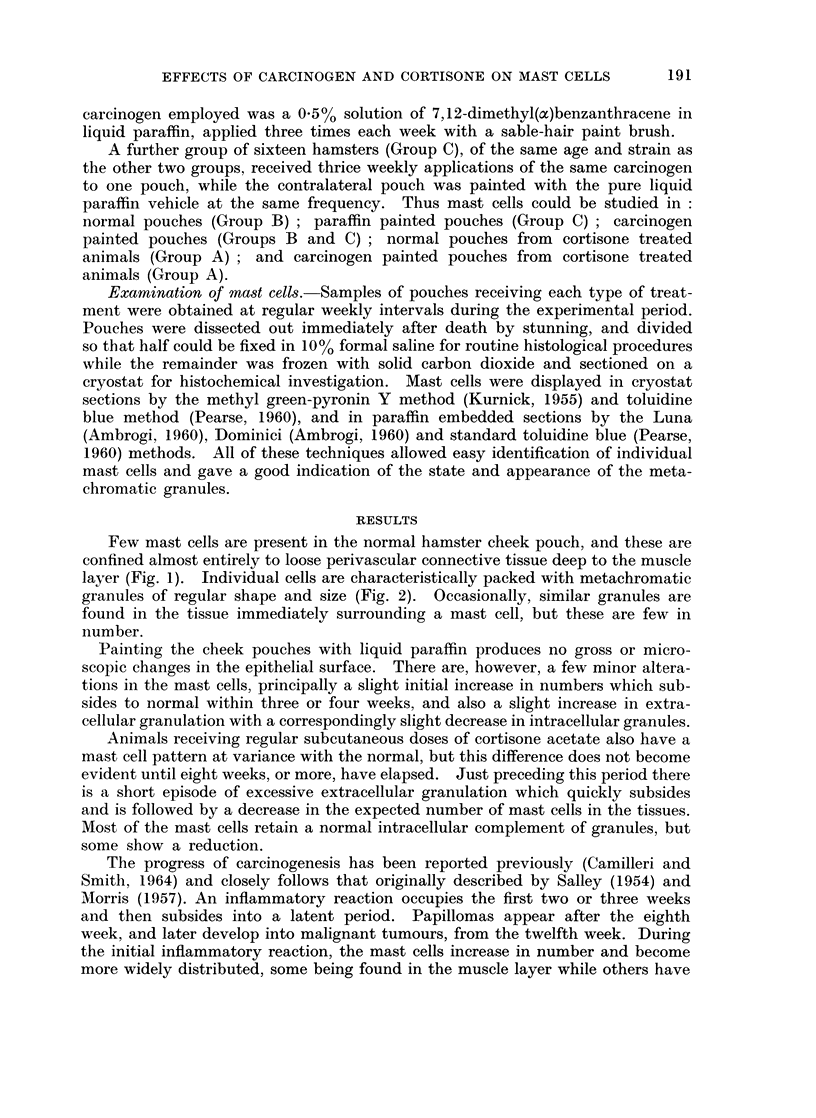

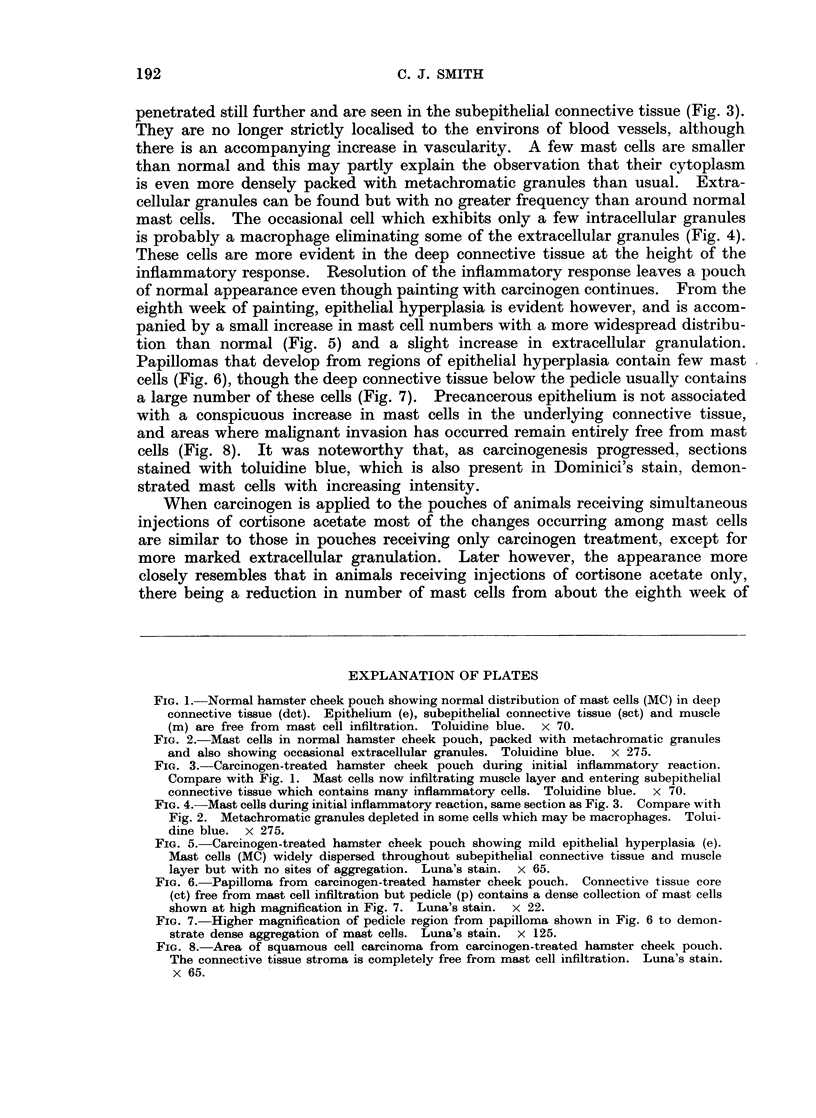

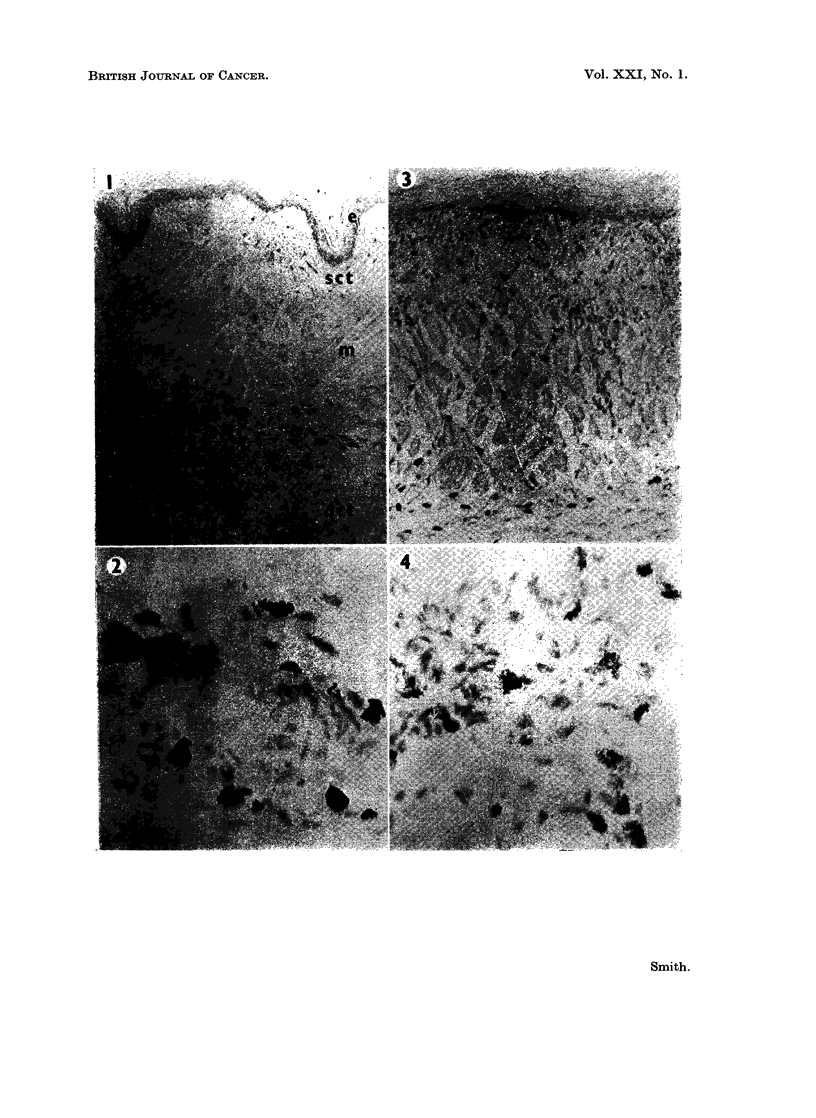

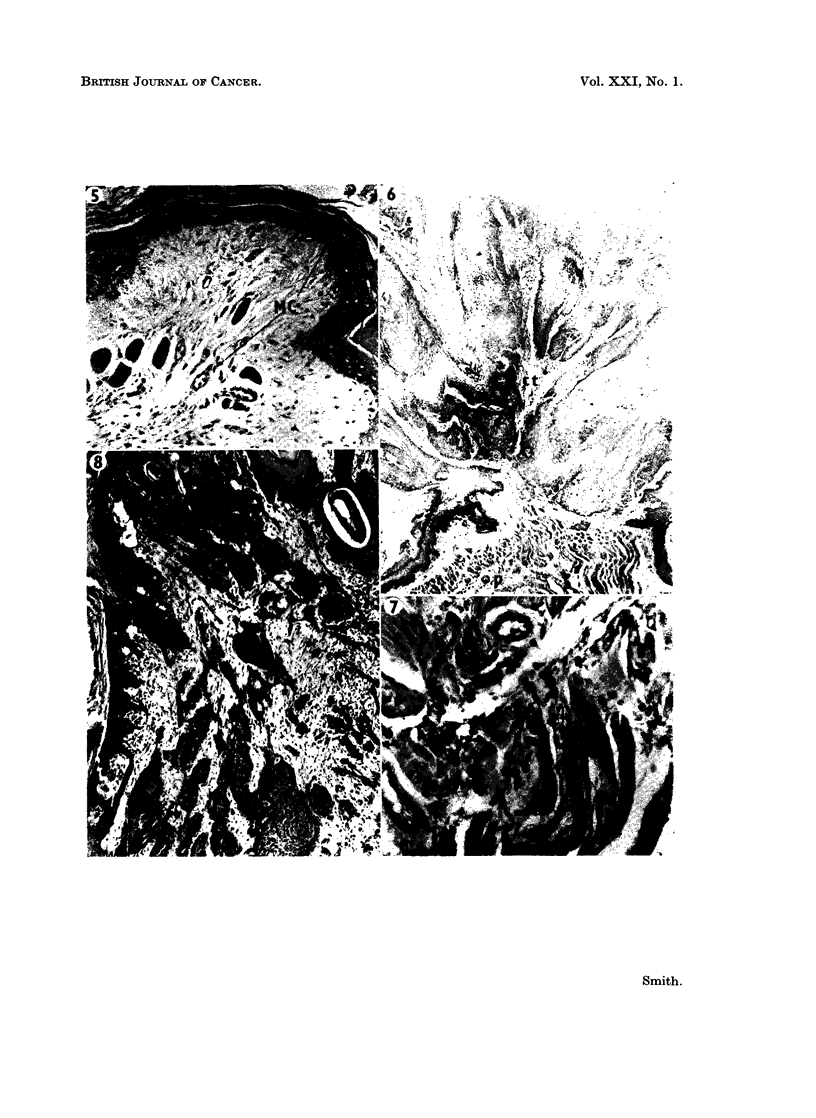

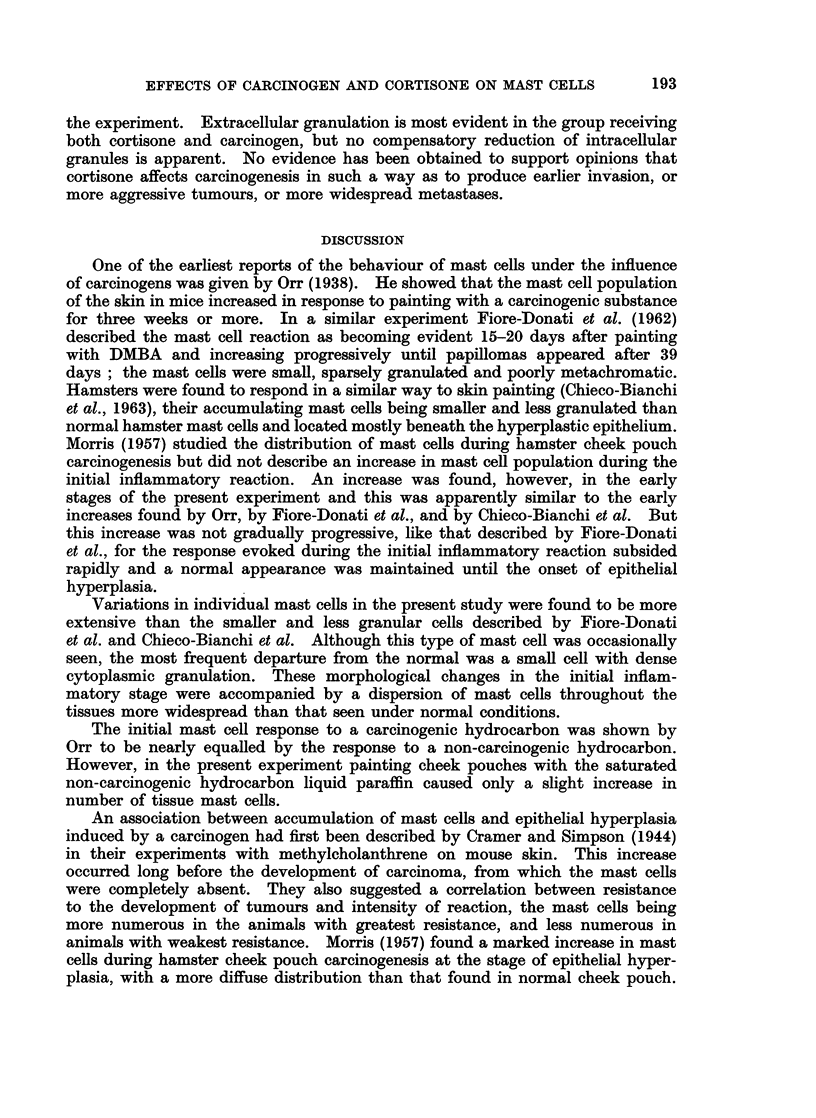

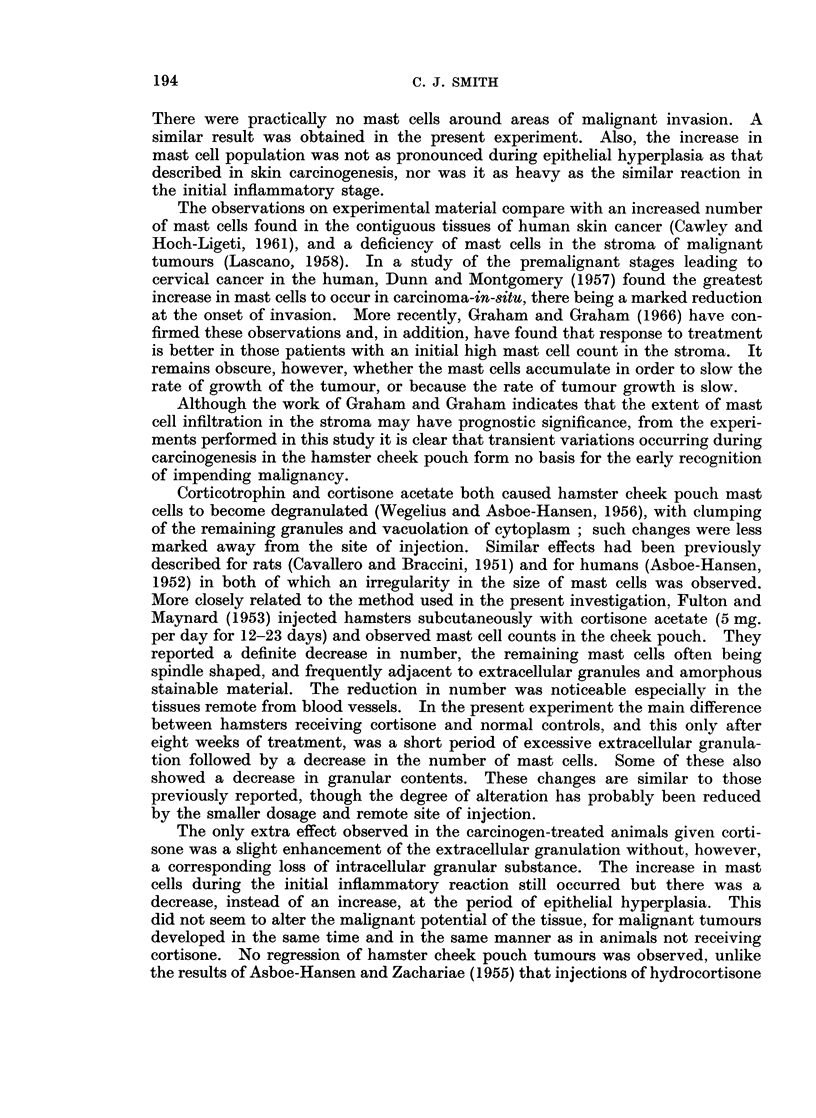

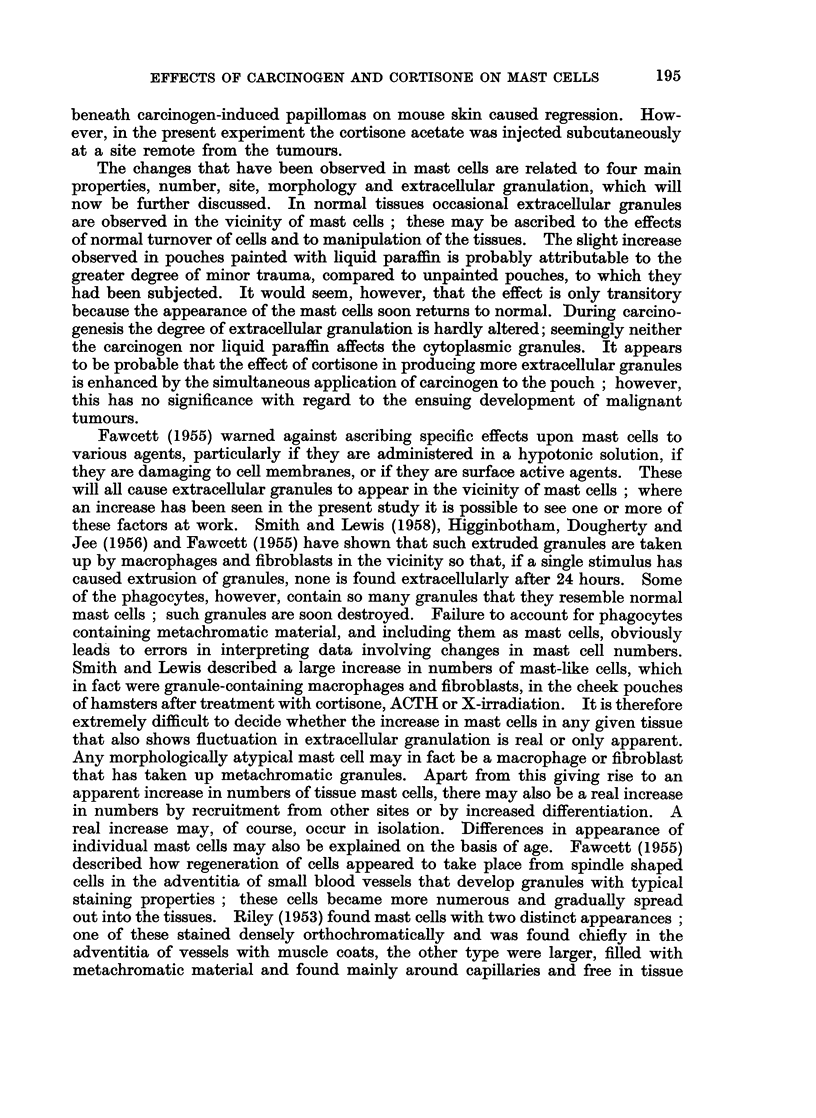

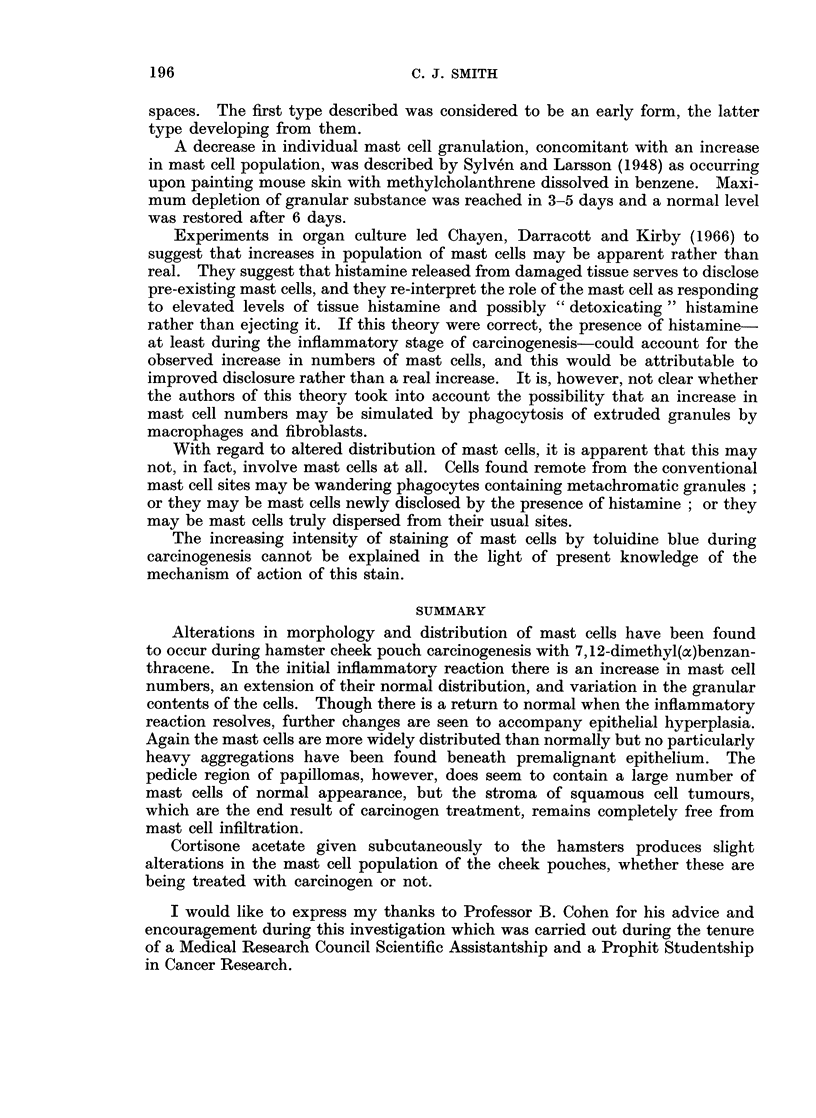

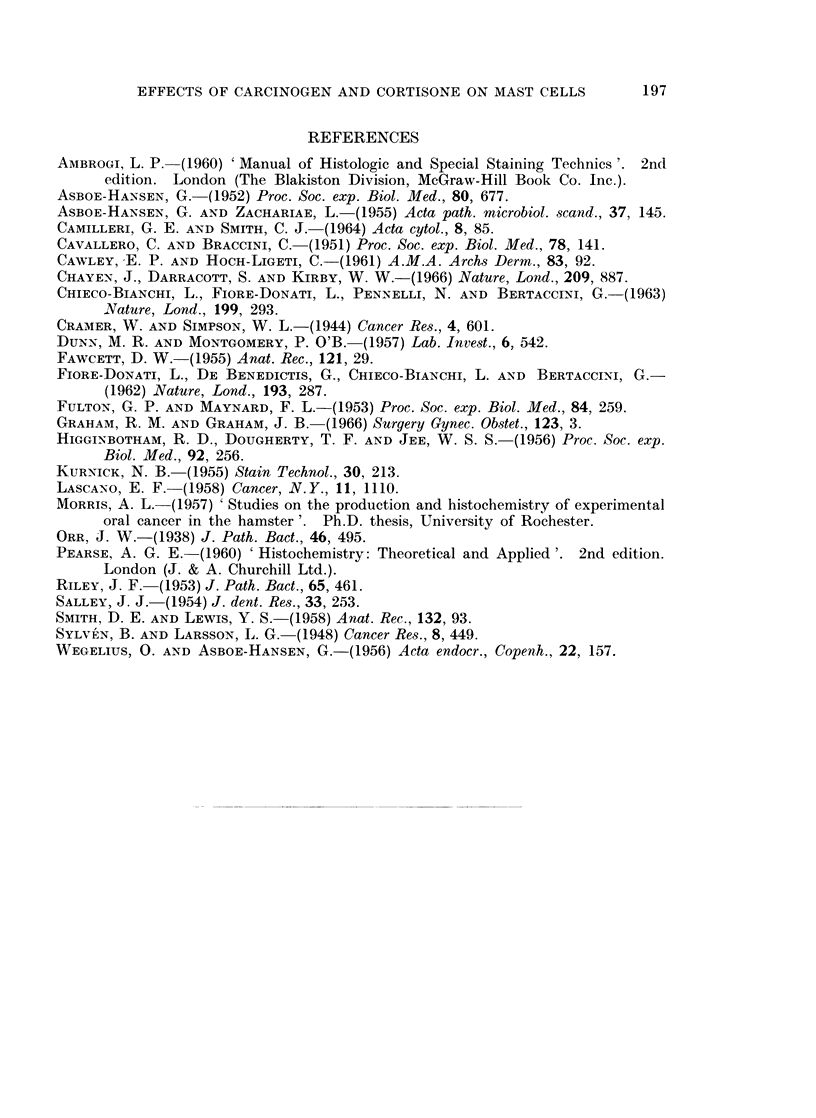


## References

[OCR_00442] ASBOE-HANSEN G. (1952). The mast cell. Cortisone action on connective tissue.. Proc Soc Exp Biol Med.

[OCR_00444] ASBOE-HANSEN G., ZACHARIAE L. (1955). Mast-cell changes induced by hydrocortisone acetate in experimental skin papillomas in mice.. Acta Pathol Microbiol Scand.

[OCR_00447] CAVALLERO C., BRACCINI C. (1951). Effect of cortisone on the mast cells of the rat.. Proc Soc Exp Biol Med.

[OCR_00452] CHIECO-BIANCHI L., FIORE-DONATI L., PENNELLI N., BERTACCINI G. (1963). MAST CELL REACTION AND 5-HYDROXYTRYPTAMINE CONTENT IN THE SKIN OF SYRIAN GOLDEN HAMSTERS PAINTED WITH 9:10-DIMETHYL-1:2-BENZANTHRACENE.. Nature.

[OCR_00450] Chayen J., Darracott S., Kirby W. W. (1966). A re-interpretation of the role of the mast cell.. Nature.

[OCR_00458] DUNN M. R., MONTGOMERY P. O. (1957). A study of the relationship of mast cells to carcinoma in situ of the uterine cervix.. Lab Invest.

[OCR_00461] FIORE-DONATI L., DE BENEDICTIS G., CHIECO-BIANCHI L. (1962). Development of mast cell reaction during chemical skin carcinogenesis of mouse.. Nature.

[OCR_00466] FULTON G. P., MAYNARD F. L. (1953). Effect of cortisone on tissue mast cells in the hamster cheek pouch.. Proc Soc Exp Biol Med.

[OCR_00468] HIGGINBOTHAM R. D., DOUGHERTY T. F., JEE W. S. (1956). Fate of shed mast cell granules.. Proc Soc Exp Biol Med.

[OCR_00472] KURNICK N. B. (1955). Pyronin Y in the methyl-green-pyronin histological stain.. Stain Technol.

[OCR_00473] LASCANO E. F. (1958). Mast cells in human tumors.. Cancer.

[OCR_00484] RILEY J. F. (1953). The relationship of the tissue mast cells to the blood vessels in the rat.. J Pathol Bacteriol.

[OCR_00485] SALLEY J. J. (1954). Experimental carcinogenesis in the cheek pouch of the Syrian hamster.. J Dent Res.

[OCR_00491] WEGELIUS O., ASBOE-HANSEN G. (1956). Hormonal effects on mast cells; studies on living connective tissue in the hamster cheek pouch.. Acta Endocrinol (Copenh).

